# Myelin sheath and cyanobacterial thylakoids as concentric multilamellar structures with similar bioenergetic properties

**DOI:** 10.1098/rsob.210177

**Published:** 2021-12-15

**Authors:** Alessandro Maria Morelli, Mariachiara Chiantore, Silvia Ravera, Felix Scholkmann, Isabella Panfoli

**Affiliations:** ^1^ Pharmacy Department, Biochemistry Lab, University of Genova, Genova, Italy; ^2^ Department of Earth, Environment and Life Sciences, University of Genova, Genova, Italy; ^3^ Experimental Medicine Department, University of Genova, Genova, Italy; ^4^ Biomedical Optics Research Laboratory, Department of Neonatology, University Hospital Zurich, University of Zurich, Zurich, Switzerland

**Keywords:** ATP synthase, cyanobacteria, myelin, oxidative phosphorylation, photosynthesis, thylakoid

## Abstract

There is a surprisingly high morphological similarity between multilamellar concentric thylakoids in cyanobacteria and the myelin sheath that wraps the nerve axons. Thylakoids are multilamellar structures, which express photosystems I and II, cytochromes and ATP synthase necessary for the light-dependent reaction of photosynthesis. Myelin is a multilamellar structure that surrounds many axons in the nervous system and has long been believed to act simply as an insulator. However, it has been shown that myelin has a trophic role, conveying nutrients to the axons and producing ATP through oxidative phosphorylation. Therefore, it is tempting to presume that both membranous structures, although distant in the evolution tree, share not only a morphological but also a functional similarity, acting in feeding ATP synthesized by the ATP synthase to the centre of the multilamellar structure. Therefore, both molecular structures may represent a convergent evolution of life on Earth to fulfill fundamentally similar functions.

## Graphical abstract

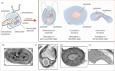

## 1. Introduction

Many axons are surrounded by myelin, a multilamellar membrane produced by specialized glial cells (i.e. Schwann cells in the peripheral nervous system and oligodendrocytes in the central nervous system). Myelin plays a pivotal role in the axon surroundings, and evidence is gathering that, as well as its insulating role, myelin also plays an unexplained neuro-trophic role, as its loss causes axonal degeneration. The latest research indicates that myelin sheath bioenergetically supports nerve conduction by speeding it up through aerobic ATP synthesis thanks to the expression of the mitochondrial machinery that carries out oxidative phosphorylation (OXPHOS) therein [1,2]. Experimental data suggest that the sheath offers bioenergetic support to the axon, conveying nutrients to the axoplasm [3–5].

**Figure 1.**
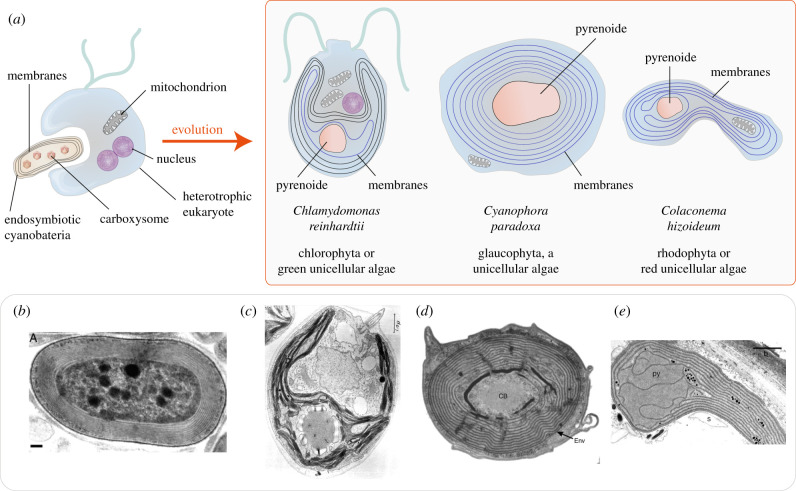
(*a*) Evolution scheme with primary endosymbiosis of cyanobacteria. The cyanobacteria with a concentric thylakoid were engulfed in three single-celled algae phyla. Organisms that were formed by this endosymbiotic process were *Chlamydomonas reinhardtii* (Chlorophyta), *Cyanophora paradoxa* (Glaucophyta; a freshwater alga) and *Colaconema hizoideum* (Rhodophyta; a red unicellular alga). In such single-celled algae, the concentric multilamellar thylakoid structure is called cyanelles. Cyanobacteria contain multiple carboxysomes which evolved in central pyrenoids in unicellular algae. (*b*–*e*) Electron microscopy (EM) images of the respective cyanobacteria. (*b*) EM image of a chlorophyll d-producing cyanobacteria (strain CCMEE 5410). Reproduced from Miller *et al*. [6], with permission from the publisher. (*c*) EM image of the unicellular green alga *Chlamydomonas reinhardtii*. Reproduced from Ohad *et al*. [7], with permission from the publisher. (*d*) EM image of a *Cyanophora* cell. Reproduced from Fathinejad *et al*. [8], with permission from the publisher. (*e*) EM image of a plastid of *Colaconema rhizoideum* containing a large pyrenoid (py) penetrated by thylakoids. Reproduced from Yoon *et al.* [9], with permission from the publisher.

**Figure 2.**
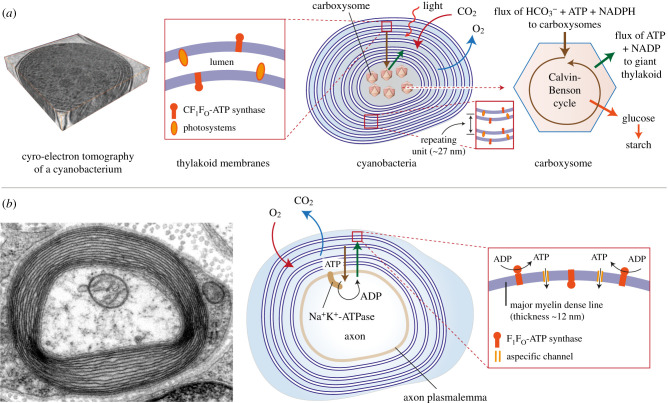
(*a*) Main gas and metabolite fluxes in cyanobacteria. A cryo-electron tomography of a cyanobacterium is shown on the left, highlighting the thylakoid membranes. Reproduced from Ting *et al*. [10], with permission from the publisher. On the right, a schematic is shown focusing on visualizing the concentric multilamellar thylakoid membranes of a cyanobacterium. On the thylakoid membrane, the presence of photosystems and CF_1_F_O_-ATP synthase is highlighted, which synthesizes ATP thanks to the proton flux generated by the photosystem complexes. On the right, a carboxysome is schematized where the reactions of the Calvin–Benson cycle take place, fed by the flux of HCO^3−^ + ATP + NADPH coming from the concentric thylakoids. (*b*) Electron microscopic image of an axon. Reproduced from the Electron Microscopy Faculty of Trinity College (Creative Commons licence). On the right, a scheme of a myelinated nerve section is shown. The insert shows the location of the F_1_F_O_-ATP synthase on the myelin membrane moved by the proton currents generated by the respiratory complexes that consume oxygen releasing CO_2_. The ATP flow is sent to the central axon through non-specific channels where the Na^+^ K^+^-ATPase hydrolyses it to ADP to keep the different ionic distribution on both sides of the plasma membrane constant and to support nerve conduction. ADP returns to myelin sheath where it is resynthesized.

Surprisingly, a striking similarity can be observed between the spiralized myelin sheath that surrounds a nerve and the concentric multilamellar thylakoid of a cyanobacterium (compare figures 1 and 2), such as the thylakoid of chlorophyll d-producing cyanobacteria (figure 1*b*) and *Prochlorococcus*, which constitute about 50% of marine cyanobacteria. These are characterized by extremely small sizes (0.5–0.7 µm) [11]. This similarity is all the more impressive when we consider that the species containing these structures are enormously distant on the evolutionary scale. Cyanobacteria are, in fact, among the main constituents of marine phytoplankton, which absorbs atmospheric carbon dioxide (CO_2_), performing primary oxygen (O_2_) production through photosynthesis, but also nitrogen fixation. The multilamellar structure was maintained when cyanobacteria evolved into plastids through endosymbiosis with single-cell plant species.

In face of morphological analogies, the functions that these structures perform appear highly diversified (i.e. chlorophyll synthesis and nitrogen fixation in cyanobacteria, and insulation in the nervous system). Nonetheless, the evidence showing that myelin is able to conduct OXPHOS renders the analogy much more stringent in terms of function. Myelin speeds up nerve conduction, aerobically producing the ATP required by the Na^+^ K^+^-ATPase pump of the axonal plasma membrane. The biochemical processes operating in both structures include the ATP aerobic synthesis by the nanomachine ATP synthase and the conversion of CO_2_ to bicarbonate by carbonic anhydrase (CA) activity. Another natural physical property these structures share is gas absorption ability, namely absorption of CO_2_ and nitrogen in the cyanobacteria, and of O_2_ in the myelin sheath. Lipids, especially neutral ones, dissolve gases about five times better than water. Thus, by comparing the lipid-rich concentric multilamellar structure of cyanobacteria thylakoids and the myelin sheath, it is possible to hypothesize that both structures may be functional to the absorption of the respective gases due to the maximization of surface area per unit volume in both systems.

## 2. Endosymbiosis of cyanobacteria

Several unicellular plants have incorporated cyanobacteria (figure 1) by endosymbiosis, forming plastids. This was the case for three species of unicellular algae: *Chlamydomonas reinhardtii* (of the phylum Chlorophyta), *Cyanophora paradoxa* (of the phylum Glaucophyta) and *Colaconema rhizoideum* (of the phylum Rhodophyta). Subsequent and complex processes of secondary and tertiary endosymbiosis also occurred. In our discussion, we will focus on the primary endosymbiosis of the three species just mentioned. It is interesting that in all three species plastids are present in the form of concentric multilamellar structures, called cyanelles, which derive by primary symbiosis from the multilamellar thylakoids of cyanobacteria and which have marked similarities with them [12].

Cyanobacteria are very ancient organisms. It is believed that they are responsible for O_2_ appearance in the Earth's atmosphere, around 2.45–2.22 billion years ago. Cyanobacteria are of enormous interest in evolution as they are the only unicellular organisms that have evolved into multicellular organisms differently from non-photosynthetic prokaryotes [13]. Evolutionary studies indicate that an endosymbiosis of cyanobacteria occurred in an ancestral eukaryotic cell, producing the typical chloroplasts of (i) green algae (see *Chlamydomonas reinhardtii*, figure 1*c*) and of plants, (ii) the restricted family of unicellular algae glaucophytes with a controversial classification (see *Cyanophora paradoxa*, figure 1*d*), and (iii) red algae (see *Colaconema rhizoideum*, figure 1*e*). The three species contain chloroplasts, called cyanelles, or very superficially developed muroplasts with particular characteristics [14].

## 3. Generality of gas absorption in biological systems

To capture O_2_, a passage from the atmospheric gaseous phase to the cytoplasmic liquid phase is necessary. Then oxygen is used by cytochrome *c* oxidase to reduce water, transferring four electrons supplied by the electron transport chain. In this way, a proton flux is formed to sustain the ATP synthesis through the nanomachine ATP synthase. Since the concentration of atmospheric CO_2_ is about 1/500 compared to that of O_2_ (0.04% versus 21%), it is evident that CO_2_ capture is more difficult than that of O_2_. This discrepancy in the concentrations of the two gases obviously also occurs in sea water, as the CO_2_ dissolved is only 70 ppm. Therefore, it is understandable that photosynthesis is more challenging for marine autotrophs [15] compared to terrestrial plants. In order to be able to absorb CO_2_, cyanobacteria possess a CO_2_-concentrating mechanism which has been extensively studied [16]. It has been ascertained that for the CO_2_ capture by cyanobacteria, an apparatus performing a ‘sponge’ effect is required. This structure could be represented by the lipid-rich multilamellar thylakoid membrane system. Similarly, myelin has been proposed to carry out O_2_ capture [1,2] due to the multilamellar lipid structure since brain tissues do not express proteins able to accumulate O_2_, such as myoglobin in muscle. Moreover, neutral lipids, normally present in membranes, such as cholesterol and waxes, bind gases better than water [17,18]. Even in plants, the first structure that interacts with atmospheric gases is the waxy cuticle [19], which is immediately in contact with the surrounding environment both in terrestrial and aquatic autotrophs, being the outermost layer present on both sides of the leaves. Subsequently, the extended surface development of thylakoid discs appears functional to the sequestration of CO_2_.

Another biological process requiring a well-developed surface is the phototransduction process both to capture O_2_ and light. In fact, since 2008, it was demonstrated that rod outer segment discs are a site of extramitochondrial OXPHOS [20–25]. Interestingly, such a property had been identified by pioneering studies carried out many years earlier by Carretta & Cavaggioni [26].

Moreover, it is noteworthy that mitochondrial cristae may also derive from the transfer of membranous structures from the endoplasmic reticulum to increase the membrane surface [27].

In other words, all structures involved in the gaseous exchanges require a well-developed membranous surface to guarantee maximum absorption of gas.

## 4. Light capture, photosynthesis, ATP production for glucose synthesis and CO_2_ accessibility to RuBisCo

In the thylakoid membranes of cyanobacteria, the energy boost is carried out by light absorption, and the incorporation of gaseous CO_2_ into organic compounds occurs through the ribulose 1,5 bis-phosphate carboxylase (RuBisCo) enzyme, producing two molecules of 3-phosphoglycerate. ATP is consumed both by ribulose-5-P kinase (i.e. the reaction upstream the RuBisCo activity) and glycerate-3-P kinase (i.e. the enzymatic steps immediately after the RuBisCo step).

In detail, atmospheric CO_2_ must be converted into bicarbonate, more soluble in the aqueous phase. For this step, thylakoid membranes express high amount of CA. Afterwards, bicarbonate produced in the thylakoid is transferred to the carboxysomes, where CA reconverts bicarbonate in gaseous CO_2_ (figure 2*a*) [16]. This apparent futile cycle is necessary to allow the transport of CO_2_ from the atmosphere to the carboxysome, where it is incorporated to 1,5 bis-phosphate ribulose by the RuBisCo. In this phase, the dispersion of gaseous CO_2_ is prevented by the gas-impermeable membranes of the carboxysome [28]. Notably, unicellular algae pyrenoid contains the same molecular structures of carboxysomes (figure 1), providing evidence of the evolution of cyanobacteria carboxysome in monocellular algae pyrenoids [29].

For CO_2_ incorporation into organic compounds, a very high RuBisCo concentration is required, as it displays a modest catalytic efficiency. For this reason, carboxysomes, dense nuclei of RuBisCo, are found at the centre of the concentric multilamellar structure (figure 1). Apart from that, RuBisCo is the most abundant protein in the biosphere because it is highly concentrated in cyanobacteria, plants and even animals. Moreover, the catalytic efficiency of marine cyanobacteria RuBisCo is three times higher than that of terrestrial cyanobacteria [30].

Therefore, it is clear that CA plays a pivotal role in CO_2_ incorporation into organic compounds, as suggested by the expression of a new subclass of CA in cyanobacteria, highlighting and further confirming its important role in the geo-cycling of CO_2_ [31]. Apart from that, CA is also contained in mitochondria [32] to convert CO_2_ released by the Krebs cycle into bicarbonate. Interestingly, CA is also expressed in the myelin sheath [33], confirming the active role of this structure in the aerobic metabolism management. Moreover, Brion *et al*. [34] have shown that upregulation of the isoenzyme CA IV in myelin results in stabilization of its structure and less susceptibility to seizures.

## 5. Cyanobacteria thylakoids and cyanelles: concentric multilamellar membranes transporting metabolites to feed the Calvin–Benson cycle

The comparison between multilamellar thylakoids of cyanobacteria with myelin sheath shines the spotlight on precise structural and functional analogies. For example, the CF_1_F_o_-ATP synthase [35] (C stands for chloroplasts) is very similar to the ATP synthase expressed in mitochondria and in other membranous structures performing OXPHOS, such as myelin [36] and rod outer segment disc [22]. ATP synthase was well evidenced in the cyanelle-like structure of the cyanobacterium *Synechococcus* [37].

In figure 2*a*, the processes of gas absorption/release and the metabolite flow occuring in a cyanobacterium are schematized. The left panel shows the macromolecular complexes expressed in thylakoid membranes and involved in light absorption, the first step of photosynthesis. In this site, photosystems produce a proton flux, necessary for ATP synthesis by the CF_1_F_O_-ATP synthase. Part of this energy production plays a pivotal role of the CO_2_ conversion in bicarbonate by the CA activity [38], representing a link between the light absorption and the CO_2_ incorporation in organic compounds.

Notably, since RuBiSco is sensitive to O_2_, reaction involving CO_2_ incorporation into organic compounds must be carried out in a different site with respect to that which houses photosynthesis and the related O_2_ production. In fact, O_2_ can induce an alternative oxygenation activity of RuBisCo, activating the phosphoglycolate cycle and preventing glucose synthesis through the Calvin–Benson cycle. [39]. Therefore, metabolites produced by the first phase of photosynthesis, such as bicarbonate, NADPH and ATP, pass through multiple layers of thylakoids to carboxysomes, at the centre of the cyanobacterium, where the Calvin–Benson cycle occurs. Moreover, it is evident that the transfer of these three metabolites is a crucial step since the existence of non-selective channels of 1.3 nm diameter in the thylakoid membranes has been demonstrated in *Cyanophora paradoxa* (figure 1*d*) [40]. Interestingly, the protein sequence of these channels is homologous with the voltage-dependent anion channels (VDAC), which are expressed in mitochondria and other membranous structures [41–43]. Although the channel diameter of the mitochondrial VDAC is 0.32 nm, it has been reported that the VDAC oligomerizes forming tetramers [44]. This allows us to hypothesize that the union of the C-terminal beta-barrel end with the N-terminal, repeated four times, could form a ring with a diameter of around 1.24 nm, compatible with the diameter of thylakoid channels [40]. We fully acknowledge that this hypothesis requires confirmation.

Interestingly, the green unicellular alga *Chlamydomonas reinhardtii* displays channels connecting the thylakoid stacks and the pyrenoid [45], conveying bicarbonate, NADPH and ATP to the central pyrenoid, feeding the Calvin–Benson cycle. This confirms the need for a clear physical separation between the photosynthesis and related O_2_ development and the Calvin–Benson cycle site.

## 6. Galactolipids stabilize multilamellar structures

The formation of multilamellar structures is hindered by electrostatic repulsion induced by the negative charges of the phospholipid orthophosphoric residues on both sides of all the multilamellar structures. To overcome this repulsive force, a decisive role appears to be played by galactolipids, which dominate in the plants thylakoids [46], and in all multilamellar structures examined here. Galactolipids make up about 70% of the lipids in the cyanobacteria thylakoids [47], up to 80% in plant thylakoids [48] and about 30% in myelin [49]. The low galactolipid concentration in the myelin sheath is compensated for by the high content of neutral lipids (20%), mainly represented by cholesterol [50]. Moreover, the multilamellar structures′s biosynthesis and functionality is heavily compromised by the ablation of the gene that synthesizes galactolipids [51].

As pointed out by Latza *et al*. [52], galactolipids that counteract the repulsive electrostatic force depend on the glucidic residues of galactolipids, which protrude towards the aqueous phase adhering to both the membrane and form the non-covalent saccharide bonds between the two galactoside residues. Latza *et al*. [52] highlighted that their results ‘indicate that glycolipid-mediated membrane adhesion is a highly abundant phenomenon and therefore potentially of great biological relevance’.

## 7. Myelin sheaths synthesize ATP to sustain and speed up nerve conduction

Recently, it has been shown that there is a liquid layer that separates the axon from the myelin sheath. This challenges the old hypothesis that considers myelin merely to be an ‘electrical insulator’ [53]. It was also found that ATP is transported into the axon by gap junctions, of which myelin is particularly rich [54]. Thanks to the supplied ATP, the relocation of the K^+^ ion to the outside is faster than the Na^+^ influx in the axon associated with the operation of the voltage-gated channels of the respective ions. With these insights, it emerges that myelin does not actually alter the basic chemical–physical modalities of nerve conduction according to the universally accepted Hodgkin–Huxley model. [2].

Myelin concentric multilamellar structures appear very similar to the spiralized thylakoids of the cyanobacteria. Both structures contain the molecular machinery for ATP synthesis, and both incorporate and release gases (CO_2_/O_2_), although the exchange direction is opposite. Thylakoids incorporate CO_2_ and release O_2_, while the myelin sheath incorporates O_2_ and releases CO_2_. Both systems are rich in the crucial CA enzyme. To exert this action, the presence of pores on myelin sheaths allowing the radial passage of ATP is crucial. Myelin is permeable to solutes, as demonstrated by the permeation of lucifer yellow [55], with the spread of glucose, deoxyglucose and lactate [56]. Apart from that, it is possible that VDAC also contributes to radial permeability in myelin, as occurred in thylakoids of cyanobacteria and cyanelles (see §6). Moreover, proteomic analyses of myelin sheath support this hypothesis since it was found that myelin is rich in all three forms of VDAC [57–59].

## 8. Conclusion and perspectives

The morphological similarities among concentric multilamellar structures of cyanobacteria and some unicellular algae with the myelin sheath—both rich in lipids—appear to respond to the common function of the first stage of the absorption of CO_2_ and O_2_, respectively. Both structures express high CA levels, and an electron transport chain associated with ATP synthesis conveys high flows of metabolites to the centre (ATP, NADPH and bicarbonate in thylakoid structure and ATP in myelin sheath) by a radial diffusion through VDAC or gap junctions. Moreover, the superficial development of these structures is fundamental to capture O_2_ (in myelin sheath) or CO_2_ (in thylakoids). Both structures are also characterized by a metabolite flux from the periphery to the centre, represented by carboxysomes for cyanobacteria, and the axon for the myelin sheath. In the first case, metabolites sustain the Calvin–Benson cycle, while in myelin, the transported ATP sustains axonal conduction. Therefore, a unifying criterion emerges: to achieve substrate delivery in a central area, the efficient solution is to pass metabolites radially to the centre through multilamellar membranes, a process that is ensured by the non-specific VDAC-like pores in cyanobacteria, the existence of real channels in the species *Chlamydomonas reinhardtii* [45], and connexin in the myelin sheath [54].

This functional homology is impressive in that it concerns structures that have radically different origins. The myelin sheath derives from plasma membrane protrusions of oligodendrocytes in the central nervous system and Schwann cells in the peripheral nervous system. By contrast, the thylakoid membranes of cyanobacteria and equivalent structures in unicellular algae reflect endosymbiotic events between cyanobacteria and heterotrophic eukaryotic cells, thought to have taken place a billion years ago.

Apart from that, *in silico* simulation of the dispersion of lipids shows the spontaneous formation of multilamellar lipid vesicles [60], supporting the idea that the multilayer structure is generically stable. It is interesting that glycolipids contribute to the stability of the multilamellar structures by creating non-covalent saccharide bonds with the glucidic residues that protrude from the membrane [52]. Consideration should also be given to the fact that galactose is the predominant residue and that galactolipids are present in significant quantities in the thylakoids of cyanobacteria, in the cyanelles of unicellular algae and in myelin.

An artificial phospholipid multilamellar structure was also created using polylysine interposed between lipid layers. Simulating the structure of the myelin sheath, polylysine appeared to play a role similar to that of myelin basic protein [61]. It has also been shown that enzymes linked to the membrane in overlapping artificial phospholipid multilamellar structures linked together by polylysine have a high catalytic efficiency and the products of enzymatic activity diffuse between the layers [62]. Artificial concentric multilayer reactors have recently been built which have close similarities to multilamellar concentric thylakoids and the myelin sheath [63].

Also, concentric multilamellar structures are produced in the surfactant, which is the crucial element for good O_2_ absorption by the pulmonary alveoli. [64,65]. Similar structures are also present in the form of the lamellar body in the outermost layer of the pulmonary epithelial cells [66,67].

In conclusion, convergence is a common evolutionary occurrence when a specific function is to be achieved, and in this case morphological similarity may also imply a similar function.

## Data accessibility

This article has no additional data.
